# ACCEPTABILITY OF PODCASTS TO ENHANCE WELLNESS SKILLS AMONG OLDER ADULTS IN AN UNDERSERVED COMMUNITY

**DOI:** 10.1093/geroni/igad104.2806

**Published:** 2023-12-21

**Authors:** Mary Janevic, Rebecca Lindsay, Robin Brewer, Yolanda Hill-Ashford, Rebeca Guzman, Sheria Robinson-Lane, Afton Hassett, Susan Murphy

**Affiliations:** University of Michigan School of Public Health, Ann Arbor, Michigan, United States; University of Michigan School of Public Health, Ann Arbor, Michigan, United States; University of Michigan School of Information, Ann Arbor, Michigan, United States; Detroit Health Department, Detroit, Michigan, United States; Detroit Health Department, Detroit, Michigan, United States; University of Michigan School of Nursing, Ann Arbor, Michigan, United States; University of Michigan Medical School, Ann Arbor, Michigan, United States; University of Michigan Medical School, Ann Arbor, Michigan, United States

## Abstract

Podcasts convey information in an accessible, engaging, and memorable way, yet are seldom incorporated into behavioral interventions for older adults. We developed a podcast series as a component of the RESET intervention (Re-Engaging in Self-care, Enjoying Today), the subject of an ongoing NIH-funded randomized controlled trial in Detroit, Michigan. In RESET, community health workers teach self-management and resilience-building skills to small groups of primarily African American older adults, to address challenges to well-being and functioning caused by the COVID-19 pandemic. Participants listen to six podcast episodes, accessible by website, app, or IVR/dial-in, which feature an older-adult host from the community interviewing other community members and topic experts. Each episode is then discussed in the corresponding intervention session. The goal of the current study was to evaluate the first three podcast episodes (Power of Positivity, Eating for Well-being, and Stability in Strength) in a convenience sample of 8 older adults and agency staff from our trial’s Community Advisory Board. Using 5-point Likert scales, all respondents strongly agreed or agreed that the episodes were enjoyable, relevant, and effectively taught key wellness skills. Podcast strengths identified by respondents included: warm interactions between host and guests, motivational tone, and compelling personal stories. Areas for improvement included the need to represent additional perspectives. These data suggest that podcasts are an acceptable way to provide health information to older adults. The ongoing RESET trial will allow us to determine the extent to which podcasts can help bring about behavior change and functional improvement.

